# 

**Published:** 1861-07

**Authors:** 


☞ WESTERN DENTAL SOCIETY.—Owing to the troubled state of our country, it is decided best not to hold a meeting of the Society this year. By order of the President.

S. DUNHAM, *Cor. Sec. of W. D. S.*

☞ INDIANA STATE DENTAL ASSOCIATION.—The next semi-annual meeting, which was set for the second Tuesday of July, proximo, has been postponed, and will be held in the city of Lafayette, on the *second Tuesday of January*, 1862, at which time there will be a general announcement to the profession throughout the State.

In taking the responsibility of making the above change, the officers of the association, with the advice of several members who were consulted on the subject, have acted with a view to the interests of the association, and they trust the postponement will meet the approval of every member. Owing to the present distracted state of our country and the preoccupation of the public mind by the more exciting subject of war, and our nation's troubles, it was feared that the interest of the profession would not be sufficient, at the present time, to secure a full attendance. A large attendance upon the next meeting is particularly desirable, inasmuch as in January last, through a misunderstanding as to the time of meeting, comparatively few of the members were present, and in consequence much important business was deferred.

The Secretary improves this opportunity to request all those whom this circular reaches to furnish him, as soon as convenient, with the names and addresses of all respectable practitioners of dentistry in their vicinity, together with those of all practitioners who feel a good degree of interest in our specialty. Those who will be kind enough to forward such lists will please address the undersigned, at Terre Haute, Indiana. By order of P. G. C. HUNT, Pres't.

S. B. SMITH,

*Rec. and Cor. Sec. Ind. State Dent. Ass'n.*

---

POSTPONEMENT.—In pursuance of the general expression of desire to that effect, from the officers and members of the American Dental Association, the President, Dr. Atkinson, authorizes an announcement of the *postponement* of the regular meeting for one year.

The association will, therefore, meet in the city of Cleveland, O., upon the last Thursday of July, 1862, at 10 o'clock A. M. W. MUIR ROGERS, *Cor. Sec'y.*

### DENTAL REGISTER,

OF THE WEST.

**Issued Monthly, at $3 a year in advance.**

DEVOTED TO THE INTERESTS OF THE DENTAL PROFESSION.

**Edited by J. TAFT and GEO. WATT.**

The Volume commences in January and closes in December.

The Register being issued monthly is always fresh, therefore indispensable to the practitioner who desires to keep posted up with the new inventions and improvements which are daily developed. Neither expense nor effort will be spared to give value and variety to its contents. Articles will be illustrated with well executed engravings, whenever they will add to the interest or assist in explanation.

Its extensive circulation and frequency of issue makes it the BEST DENTAL ADVERTISING MEDIUM in the United States.

☞ CONTRIBUTIONS SOLICITED.

Address Original Communications to **Geo. Watt, Xenia, O.**

Exchanges to **J. Taft**, or **Dental Register, Cincinnati, O.**

Business Gommunications, to

**JNO. T. TOLAND, Publisher and Proprietor,**
38 West Fourth Street, Cincinnati, O.

Communications must reach the Editor as early as the 15th, to insure insertion in the next issue.

